# Progesterone Promotes In Vitro Maturation of Domestic Dog Oocytes Leading to Successful Live Births

**DOI:** 10.3390/life12111778

**Published:** 2022-11-03

**Authors:** Yumin Qin, Shenjiong Feng, Min Zheng, Xiaojuan Liu, Jianping Zhao, Qintao Zhao, Junhua Ye, Jidong Mi, Yougang Zhong

**Affiliations:** 1 College of Veterinary Medicine, China Agricultural University, Beijing 100193, China; 2 Beijing SINOGENE Biotechnology Co., Ltd., Beijing 102200, China; 3 Nanchang Police-dog Base of the Ministry of Public Security of PRC, Nanchang 330100, China

**Keywords:** canine, progesterone, oocyte in vitro maturation

## Abstract

Gene-edited dogs are promising models for biomedical research because they have hundreds of genetic diseases that are similar to humans. A common method for producing gene-edited dogs is assisted reproductive technology (ART) using in vivo oocytes or embryos, but it is much more inefficient and has a higher cost. ART for dogs has lagged mostly because of the lack of an efficient in vitro maturation system. Because early maturation of canine oocytes occurs in follicles with extremely high concentrations of progesterone (P_4_), we hypothesize that P_4_ has an important role during maturation. In this study, we obtained ovaries of female dogs and collected cumulus–oocyte complexes, which were cultured in vitro in microdrops containing different P_4_ concentrations (0, 10, 40, 100 or 200 µg/mL). We found that 40 µg/mL P_4_ produced the highest oocyte maturation rate (29.7% ± 7.1%, *p* < 0.05). We also evaluated the quality of in vitro matured oocytes by in vitro fertilization and single-cell RNA sequencing, and both indicated an improvement in oocyte developmental potential. In conclusion, we successfully obtained the first live dogs using in vitro matured oocytes by adding P_4_ to optimize the in vitro maturation system of canine oocytes, and established a new and low-cost method to produce dogs via in vitro maturation and in vitro fertilization.

## 1. Introduction

Transgenic and genome editing models have made a tremendous contribution to biological research and evaluation of drugs. Gene-edited animal models provide a powerful tool for studying the pathogenesis, pathological process, prevention and treatment of human diseases. The recent development of genome editing technology has allowed researchers to alter DNA sequences at chosen genomic loci, resulting in a wide variety of gene-edited animal models [1]. Currently, gene-edited animal models for human disease research mainly include rodent models represented by mice and rats, and large animal models represented by pigs, dogs and non-human primates [2]. Dogs are one of the earliest domesticated animals because they live in the same environment as humans, and their responses to environmental changes and physiological changes are close to those of humans [3]. Dogs are an ideal animal model for studying human diseases because more than 450 diseases have been reported, and most canine genetic diseases (approximately 360) have clinical and molecular counterparts in humans, with almost twice as many as those in other animals [4,5,6]. However, there are too few existing canine disease models to meet the requirements of scientific research.

At present, gene-edited dogs are usually prepared by cytoplasmic injection of fertilized oocytes and somatic cell nuclear transfer [7,8]. Both of these require surgical flushing of fertilized oocytes or matured oocytes in vivo from donor dogs. Because of the unique reproductive physiological characteristics of dogs, the oocytes discharged still need to mature for 72 h in the fallopian tube [9]. Therefore, before obtaining these original materials, monitoring estrus and detecting hormones of dogs are necessary, which are time-consuming and expensive.

Assisted reproductive technology (ART) for dogs could play a critical role in the creation of gene-edited dog models for evaluating new drugs to eradicate heritable diseases and the preservation of endangered canid species. However, ART for dogs has lagged far behind that for other species [10]. To date, puppies from in vitro fertilization (IVF) have only been obtained with in vivo matured (IVV) dog oocytes [11]. Saikhun et al. [12], England et al. [13] and Rodrigues et al. [14] carried out IVF using in vitro matured (IVM) dog oocytes, but no live births were achieved. To date, no offspring have been produced using dog oocytes matured in vitro by IVF or by somatic cell nuclear transfer. In the characteristic reproductive physiology of dogs, the oocytes from canine follicles are still in germinal vesicle (GV) stage, and they still need 54–72 h of maturation in the oviduct to reach metaphase II (MII) stage and have the ability to fertilize [9]. These features make dog oocytes more difficult to mature in vitro, and have greatly limited the development of canine ART. Therefore, finding an effective in vitro maturation system is important for advancing ART for canine species.

In canids, factors that trigger the resumption of oocyte meiosis are currently unknown, with research hampered by the lack of an in vitro maturation system and the low maturation rate of dog oocytes in vitro. Previous studies showed that early maturation of oocytes occurred in follicles in the presence of extremely high concentrations of progesterone (P_4_), which were approximately 1000 times higher than that of plasma [15]. Meiotic resumption of dog oocytes was completed in the oviduct when the P_4_ concentration of plasma reached 20–40 ng/mL [16]. In addition, the expression level of the progesterone receptor (PR) in oviduct epithelium increased after ovulation and was maintained at high levels for 7 days [17], suggesting that there are high P_4_ concentrations in the oviduct during this time. Aglepristone (PR antagonist) treatment delayed meiotic resumption of ovulated oocytes and inhibited progression to the metaphase II (MII) stage [18]. Several studies reported that P_4_ had a positive effect on in vitro maturation of canine (20 μg/mL) [19] and other mammalian oocytes, such as bovine (100 µM) [20,21]. However, the concentrations of P_4_ mentioned in these reports were diverse and may reflect results from oocyte collection at different stages of the estrus cycle. According to these reports, canine oocytes have been in a high concentration of P_4_ in vivo from follicles to fallopian tubes, and a series of in vitro experiments have proved that the addition of P_4_ in the in vitro maturation system is conducive to the maturation of oocytes. Therefore, we hypothesize that P_4_ is a key factor in the maturation of dog post-ovulatory oocytes.

Single-cell RNA sequencing (scRNA-seq) is an outstanding tool to identify differentially expressed genes in oocyte maturation to investigate the critical genes involved in oocyte maturation [22], oocyte viability and fertilization [23]. Recently, scRNA-seq has been used in studying the gene expression patterns between IVM oocytes and IVV oocytes from mice of advanced reproductive age, indicating some potential new targets to improve the maturity [24]. Therefore, in our study, scRNA-seq was carried out to analyze transcriptome changes between IVM oocytes and IVV oocytes from canines.

Here, we aimed to investigate the effect of P_4_ on IVM canine oocytes. In this study, we report that supplementation of P_4_ (40 µg/mL) is beneficial for oocytes maturation efficiency and quality, and we successfully obtained live birth of IVM-IVF dogs by optimizing the IVM culture system.

## 2. Methods

### 2.1. Animals

Domestic female beagle dogs aged 2–5 years were selected for this study. Seven female beagle dogs were used as oocyte donors, and four female beagle dogs were used as surrogates. Five male beagle dogs aged 2–5 years were used as semen donors. All experiments involving dogs were guided under a protocol approved by China Agricultural University in accordance with the guidelines of the Animal Care Committee of China Agricultural University (Approval No: AW21401202-2-2), and the Animal Care and Use Committee of Beijing SINOGENE Biotechnology Co., Ltd. All efforts were made to minimize suffering of the dogs.

### 2.2. Chemicals

Unless otherwise stated, all chemicals used in this study were purchased from Sigma-Aldrich (St. Louis, MO, USA).

### 2.3. Collection of Ovaries and Oocytes

Ovaries were obtained from various breeds of healthy domestic bitches at random ages of anestrus and metestrus via routine ovariohysterectomy at the China Agricultural University Veterinary Teaching Hospital. In brief, propofol was used to induce anesthesia, and isoflurane was used for continuous anesthesia. An incision was made on the abdominal midline, and then ovaries were exposed and removed. Dissected ovaries were transported in 38 °C saline solution (0.9% sodium chloride with 1% penicillin and streptomycin) within 3 h. Cumulus–oocyte complexes (COCs) were released by slicing the ovarian cortex with a scalpel blade. After three washes in TCM199 (containing 10% fetal bovine serum; Thermo, Carlsbad, CA, USA), oocytes with a diameter greater than 110 µm and with three or more layers of compact cumulus cells (CCs) were transferred to 60-mm dishes containing 100-µL microdrops of in vitro maturation medium covered with mineral oil for in vitro maturation.

### 2.4. In Vitro Maturation of COCs and Assessment of Oocyte Maturation

The COCs were randomly allocated into five groups of 40–50 oocytes for culture under different conditions. The basal medium was TCM199 (12350; Thermo, Waltham, MA, USA) supplemented with 10% fetal bovine serum, 2 IU luteinizing hormone (human pituitary, L6420), 8 IU follicle-stimulating hormone (human pituitary, F4021), 1 mM cysteine, 2 mM sodium pyruvate and 1% penicillin/streptomycin. We first tested the effect of low (control, 0 µg/mL), intermediate (200 µg/mL) and high concentration (1000 µg/mL) P_4_ supplementation. The result showed that if the dosage was greater than 200 µg/mL, the maturity rate would decrease (data not shown). Therefore, we chose to set a series of concentrations between 0–200 µg/mL for testing, and add 0, 10, 40, 100 or 200 µg/mL P_4_ (P7556) to the treatment groups, respectively. The COCs were cultured in 100-μL microdroplets with 5% CO_2_ at 37 °C. Each droplet of medium contained 20 COCs, which were cultured for 72 h with changes of the medium every 24 h.

After 72 h of in vitro maturation, the COCs were incubated with 0.1% hyaluronidase (diluted in TCM199 with 10% fetal bovine serum) for 5 min, and CCs were removed by gentle pipetting. Denuded oocytes were transferred to 5 µg/mL Hoechst33342 solution (diluted in TCM199) and incubated for 5 min. The oocytes were then transferred from the staining solution to phosphate-buffered saline and washed at least three times. Staining of the nucleus was observed under an inverted Hoffman fluorescence microscope (200×; Nikon ECLIPSE Ti-U, Japan), and images were collected to determine the cell cycle phase. According to the morphology of the nucleus [25,26], the oocyte was classified as a germinal vesicle (GV), germinal vesicle breakdown (GVBD), metaphase I (MI), metaphase II (MII) or degenerated (DE). The first polar body was detected in matured oocytes. All experiments were repeated at least three times, and the results are presented as the mean ± standard deviation. IBM SPSS (Version 22.0; IBM Corp., Armonk, NY, USA) software was used for the single-factor analysis of variance test, and the least significant difference method was selected for significance analysis.

### 2.5. IVF

The IVF medium and method were modified from Nagashima [11]. Modified canine capacitation medium (mCCM) contained 107 mM sodium chloride, 1 mM magnesium chloride, 4.78 mM potassium chloride, 1.71 mM calcium chloride dihydrate, 1.19 mM potassium dihydrogen phosphate, 0.25 mM sodium pyruvate, 2.78 mM glucose, 25 mM HEPES, 0.1% phenol red and 1% penicillin/streptomycin.

Freshly collected semen were observed under an inverted phase contrast microscope to ensure that more than 90% of spermatozoa were of normal morphology and motile. Before use, 1 mL of semen were centrifuged at 100× *g* for 1 min, and the supernatant was collected in a new 15-mL centrifuge tube. A volume of 3 mL mCCM was added to the pellet, and the sample was centrifuged at 400× *g* for 5 min. This operation was performed three times. Finally, the washed pellet was resuspended in mCCM and adjusted to a final concentration of 7.5 × 10^6^ sperm/mL with mCCM, and then incubated for capacitation at 38.5 °C with 5% CO_2_ for 2–4 h. To perform IVF, 1 × 10^6^ capacitated sperm were added to oocytes (after 48 h of in vitro maturation) in modified synthetic oviductal fluid microdrops [7] and co-cultured for 14 h. After IVF, oocytes were transferred to new modified synthetic oviductal fluid droplets for culture until they stopped developing. Oocytes were cultured for up to 3 days, and the medium was changed every 2 days. Fertilized oocytes were transferred into recipients, and the remaining embryos were isolated from CCs to evaluate maturity.

### 2.6. Detection of the Ovulation Date of Recipient Dogs

The ovulation date of recipient dogs was determined by measuring serum P_4_ concentrations from serum after bloody discharge was first observed from the vagina. Blood samples (2 mL) were collected daily at the same time by cephalic venipuncture, and serum was prepared by centrifugation at 300× *g* for 20 min. Serum P_4_ concentrations were assayed using an ichroma cProgesterone kit (i-CHROMA, Chuncheon-si, Korea) [26]. The first day that the serum P_4_ concentration reached 4.0–7.5 ng/mL was considered the ovulation day.

### 2.7. Embryo Transfer and Detection of Pregnancy

Two- to eight-cell embryos were placed in the ampulla of the recipient oviduct using a 3.5 F Tom cat catheter (Covidien, Juarez, Mexico) by laparotomy using aseptic surgical procedures, 3–4 days after the recipient serum P_4_ concentration reached 4.0–7.5 ng/mL [7]. Twenty-five days after embryo transfer, pregnancies were checked using an ultrasound scanner (MYLAB 30CV; Esaote, Genova, Italy). After the initial confirmation of pregnancy, fetal development was checked every 2 weeks by an ultrasound examination of the heart rate, movement, gestational sac, diameter, crown-rump length and biparietal diameter.

### 2.8. Preparation of Oocytes for scRNA-seq

IVV oocytes were collected by surgery 3–4 days after ovulation day. The protocol for oocyte collection was performed as previously described [7]. Briefly, as mentioned above, serum P_4_ was used to determine the ovulation day. Oocytes were collected by laparotomy using aseptic surgical procedures. An incision was made on the linea alba, and the ovaries were located caudal to the kidneys. An inverted flanged bulb steel needle was inserted into the fimbria of the oviduct, and a 24-gauge intravenous (IV) catheter was inserted into the isthmus of the oviduct near the uterotubal junction. The flushing medium was introduced into the oviduct through the IV catheter and oocyte flowed out. Single-cell sequencing and bioinformatics were performed by GeekGene (Beijing, China). The scRNA-seq library was constructed by following the Smart-seq2 protocol [27]. A single denuded oocyte was washed three times in phosphate-buffered saline and immediately transferred to lysis buffer (4 µL) containing 0.1 µL RNase inhibitor (Clontech, San Jose, CA, USA), 1.9 µL Triton X-100 solution (1%), 1 µL dNTP mix (10 mM) and 1 µL oligo-dT primer (5 µM), and the single cell was transferred in the lowest possible volume (≤0.5 µL). Reverse transcription was performed using 0.5 µL SuperScript II reverse transcriptase (200  IU/µL, Invitrogen, Waltham, MA, USA), 0.25 µL RNAse inhibitor (40 IU/µL, Clontech), 2 µL Superscript II First-Strand Buffer (5×, Invitrogen), 0.5 µL dithiothreitol (0.1 M, Invitrogen), 2 µL Betaine (5 M), 0.06 µL MgCl_2_ (1 M) and 0.1 µL template-switching oligos (100 µM). In total, we collected 12 mature oocytes, 4 IVV oocytes (named IVV1, IVV2, IVV3, and IVV4), 4 IVM oocytes with 40 μg/mL P_4_ (named IVMP41, IVMP42, IVMP43, and IVMP4) and 4 IVM oocytes without P_4_ (named IVMCK1, IVMCK2, IVMCK3, and IVMCK4). IVV1 was excluded because of a low comparison quality (11.0%), and IVMCK1 and IVMP41 were removed from the study because they were from different batches of ovaries. To control variables, we only selected three samples from each group (IVV, IVMP4 and IVMCK groups) for comparative analysis. Reverse transcription was carried out at 25 °C for 5 min and at 42 °C for 60 min, followed by 50 °C for 30 min and 72 °C for 10 min. Polymerase chain reaction (PCR) preamplification was performed using the KAPA HiFi HotStart Ready MIX (KAPA Biosystems, Wilmington, USA) with 22 cycles of PCR, and the IS PCR primer (PCR primer in the amplification step after reverse transcription) was reduced to 50 nM. These 22 cycles comprised 4 cycles at 98 °C for 20 s, 65 °C for 30 s and 72 °C for 5 min, followed by 18 cycles at 98 °C for 20 s, 67 °C for 15 s and 72 °C for 5 min, with a final cycle at 72 °C for 5 min. Amplified samples were then purified twice with 0.8× Ampure XP beads (A63882; Beckman, Indianapolis, IN, USA).

A library was constructed using the enriched cDNA fragments, which were attached to C1 beads using KAPA Hyper Prep Kits (KK8505, KAPA Biosystems). The New England Biolabs U-shape adaptor was used for ligation. Libraries were sequenced to generate 150-bp paired-end reads on an Illumina X-Ten platform.

### 2.9. Short Tandem Repeat Polymorphism Identification

Sixteen microsatellites, namely PEZ2, PEZ3, PEZ5, PEZ6, PEZ8, PEZ12, PEZ15, PEZ20, PEZ21, FH2010, FH2054, FH2079, FH2132, FH2611, VWFX and DA, were chosen to determine kinship.

### 2.10. Quality Control and Summary of Alignment

Quality control was performed for the raw reads obtained from scRNA-seq experiments. Raw RNA-seq reads were trimmed of adaptor sequences and low-quality reads using cutadapt (v1.10). The stripped sequences were then aligned to the dog reference genome of CanFam3.1 using Tophat (version 2.0.13) (parameters: -i 36 -I 20,000 -p 5 -r 20 –mate-std-dev 50).

ftp://ftp.ncbi.nlm.nih.gov/g-nomes/all/GCF/000/002/285/GCF_000002285.3_CanFam3.1/GCF_000002285.3_CanFam3.1_genomic.fna.gz (accessed on 2 November 2011). The averages MapRate was 81.50% for nine samples, and an alignment summary is shown in Table S1. Reads aligned to genes were counted by cufflinks (v2.2.1). The fragments per kilobase of transcript per million (FPKM) were normalized using cuffnorm. Differentially expressed genes (DEGs) were calculated using cuffdiff [28,29,30]. Unsupervised hierarchical clustering was performed using log2(FPKM+1) across the samples.

### 2.11. Gene Ontology and Kyoto Encyclopedia of Genes and Genomes Analysis

We performed gene classification and enrichment analyses for Kyoto Encyclopedia of Genes and Genomes (KEGG) [31] and Gene Ontogeny (GO) [32] pathways using TopGO (v2.24.0) and the ontology-based tool clusterProfiler, respectively.

### 2.12. K-Means Analysis

K-means clustering aims to partition n observations into k clusters in which each observation belongs to the cluster with the nearest mean, serving as a prototype of the cluster [33]. Nine oocytes in this study were the observation points, the origin was defined as the centroid, and the absolute value of the component was the sum of the squared distance from the observation point to the centroid of the cluster. K-means selected the clustering method with the smallest sum of squared distances (loss function) between the nine observation points and the cluster centroid as the result. We used K = 2 or K = 3 in the current analysis of DEGs.

### 2.13. Statistical Analysis

All experiments were repeated at least three times, and results are presented as the mean ± standard deviation. SPSS (Version 22.0, Chicago, IL, USA) was used for the single-factor ANOVA test. Differences among means were identified with Duncan’s tests, differences with *p* < 0.05 were considered significantly different.

## 3. Results

### 3.1. Optimization of the Canine IVM Oocyte System

**Figure 1:**
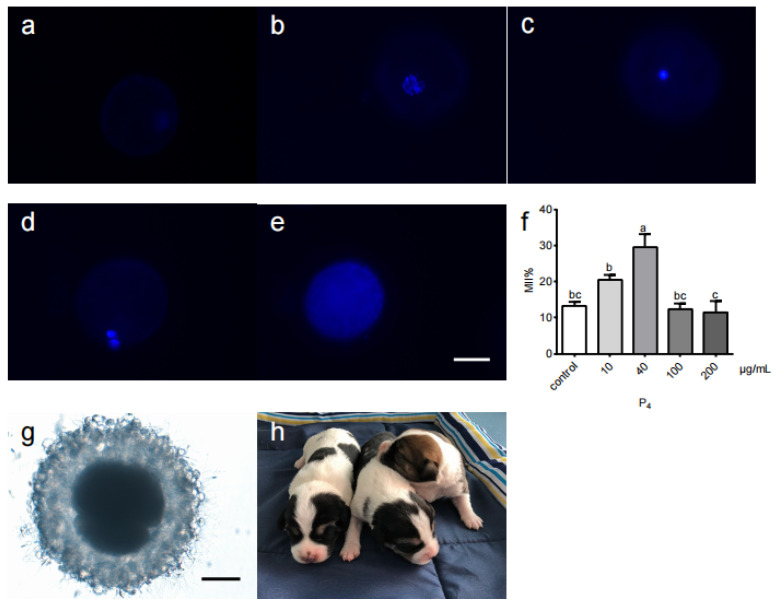
Different stages of canine oocyte development. (**a**–**e**) The oocytes were stained with Hoechst 33342. According to the nuclear morphology, it can be divided into five stages. (**a**) Germinal vesicle oocyte. (**b**) Germinal vesicle breakdown oocyte. (**c**) Metaphase I oocyte. (**d**) Metaphase II oocyte. (**e**) Degenerated oocyte. (**f**) Rate of metaphase II development for different concentrations of P_4_. Differences among means were identified with Duncan’s tests. a, b, c: in the same column, values with different superscript letters differed significantly (*p* < 0.05). (**g**) A four-cell stage embryo after in vitro fertilization produced from an in vitro matured oocyte (40×). (**h**) Three puppies derived from dog oocytes via in vitro maturation/in vitro fertilization. P_4_, progesterone. Scale bars represent (**e**,**g**) 50 μm.

The oocyte maturation rate was examined according to the morphology of the nucleus (Figure 1a–e). The highest maturation rate (29.7% ± 7.1%) was achieved with 40 µg/mL P_4_ (*p* < 0.05). High P_4_ concentrations significantly reduced the maturation rate (*p* < 0.05) (Table 1, Figure 1f).

### 3.2. Birth of IVM-IVF Puppies

Oocytes cultured in in vitro maturation medium supplemented with 40 µg/mL P_4_ were used for IVF with fresh dog sperm to produce IVM-IVF puppies (Figure 1g,h). There were 29 oocytes for IVF. Six oocytes developed into the two-cell stage. Twelve oocytes developed into the four-cell to 8-cell stage. Four oocytes developed into eight-cell stage (Table 2). The cleavage rate was 22/29 (75.9%). The representative figure of in vitro matured and in vitro fertilization canine oocytes was shown in Figure S1. Sixteen two- to eight-cell stage embryos were transferred into two recipient dogs in heat, and one became pregnant and had a natural birth of three healthy puppies (Table 3). The pregnancy rate was 25% (4/16), and the birth rate was 18.8% (3/16). The results of short tandem repeat polymorphism identification (Table S2) showed that all three puppies (Milk, Small Black, and Yellow) were derived from donor male dogs, but not the surrogate bitch. Among the three puppies, two were female and one was male. To date, two puppies are still alive, and one died of a disease.

Additionally, 29 IVV oocytes were applied for IVF. Six oocytes developed into the two-cell stage. Thirteen oocytes developed into the four-cell to the eight-cell stage. Seven oocytes developed to the eight-cell stage (Table 2). The cleavage rate was 26/29 (89.7%). Nine two to eight-cell stage embryos were transferred into two recipient dogs in heat, and one became pregnant and had a natural birth of six healthy puppies (Table 3). The pregnancy rate was 66.7% (6/9), and the birth rate was 66.7% (6/9).

### 3.3. DEG Clustering of Mature Oocytes in Vivo and in Vitro

**Figure 2:**
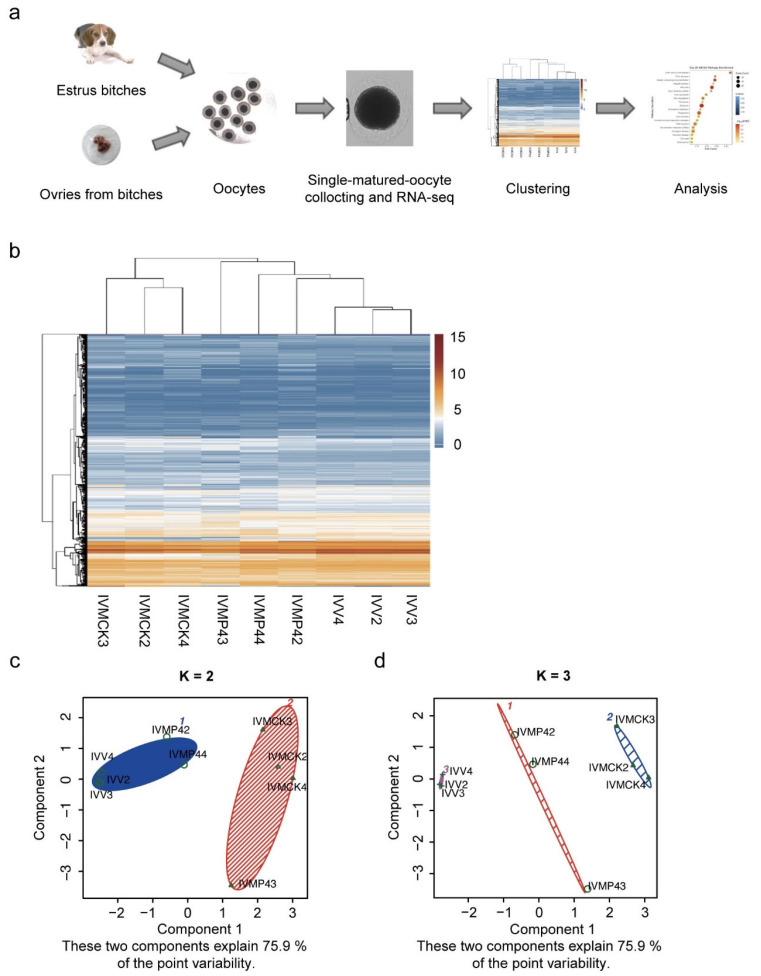
Generation of in vitro fertilization-derived dogs and analysis by single-cell RNA sequencing. (**a**) Whole process of single-cell sequencing in this study. (**b**) Heatmap of unsupervised clustering. Unsupervised hierarchical clustering was carried out using log2(FPKM+1) across the samples. The 9979 genes used for clustering were selected using 1 < maximum[log2(FPKM+1)] < 20 and a standard deviation sd[log2(FPKM+1)] > 0.3. (**c**) K-means analysis: K = 2. Nine observations were partitioned into two clusters. (**d**) K-means analysis: K = 3. Nine observations were partitioned into three clusters, and each experimental treatment group was grouped into one cluster. FPKM, fragments per kilobase of transcript per million; IVMP4, in vitro matured with progesterone; IVMCK, in vitro matured without progesterone; IVV, in vivo matured.

We performed scRNA-seq on IVM oocytes with P_4_ (IVMP4 group) or without P_4_ (IVMCK group), and IVV oocytes (IVV group) (Figure 2a). Among these samples, 9979 DEGs were divided into two groups (Figure 2b), with IVMP4 and IVV genes clustered into one group and IVMCK genes clustered into the other group. In the K-means clustering algorithm, when K = 2, IVMP42 and IVMP44 were clustered with the IVV2, IVV3 and IVV4 groups, and the observation points of IVMP2 and IVMP44 were closer to the IVV group (Figure 2c). When K = 3, the data of the three groups were separately clustered, and the observation points of the members in the group were close. However, there were differences between the groups, and the observation points (IVMP42, IVMP43 and IVMP44) for P_4_ supplementation were far from those without P_4_ (Figure 2d). These results indicated that IVM oocytes with P_4_ supplementation changed some gene expression and were closer to IVV oocytes.

### 3.4. Differences in Gene Expression of Mature Oocytes in Vivo and in Vitro

To identify differences in gene expression, we focused on DEGs. We found that IVMP4 oocytes had fewer DEGs than IVMCK oocytes (2590 versus 4366) compared with IVV oocytes. There were 1233 DEGs between the two IVM groups (IVMP4 versus IVMCK, Table 4). The IVMP4 group was more similar to the IVV group than the IVMCK group, although gene expression in these two groups was still far from the IVV group.

**Figure 3:**
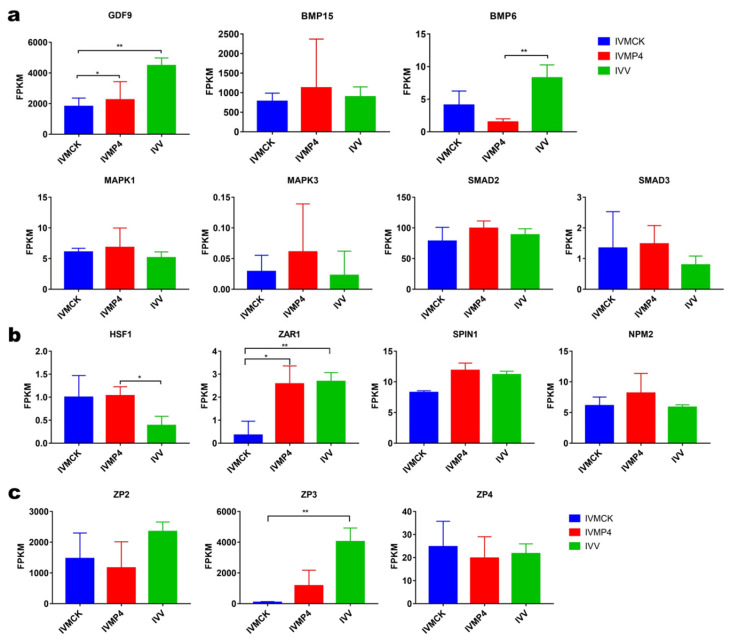
Differences in expression of functionally related genes at different stages of oocyte development. (**a**) Expression of oocyte maturation-related genes. (**b**) Expression of maternal effect genes related to early embryo development. (**c**) Expression of genes related to fertilization capabilities. IVMP4, in vitro matured with progesterone; IVMCK, in vitro matured without progesterone; IVV, in vivo matured. * *p* < 0.05 and ** *p* < 0.01.

The expression of several reported oocyte maturation-related genes, such as growth differentiation factor-9 (*GDF9*), bone morphogenetic protein-6/15 (*BMP6/15*), mitogen-activated protein kinase 1/3 (*MAPK1/3*) and small mother against decapentaplegic 2/3 (*SMAD2/3*), was similar in IVMP4 and IVV oocytes, except for *BMP6*, which had a significantly lower expression in IVMP4 oocytes than in IVV oocytes. The expression of *MAPK1/3* and *SMAD2/3* was equivalent in the IVMCK, IVMP4, and IVV groups. *GDF9* expression in IVMCK oocytes was significantly downregulated compared with that in IVV and IVMP4 oocytes (Figure 3a).

The expression of oocyte-specific genes affecting early embryonic development, such as zygote arrest 1 (*ZAR1*) and spindlin 1 (*SPIN1*), was significantly higher in canine IVMP4 oocytes than in IVMCK oocytes. However, there was no difference in nucleoplasmin 2 (*NPM2*) and heat-shock factor 1 (*HSF1*) expression between the IVMP4 and IVMCK groups (Figure 3b).

Zona pellucida glycoprotein (ZP) can be used as a molecular marker of fertilization and developmental ability, and ZPs are encoded by the *ZP1*, *ZP2, ZP3* and *ZP4* genes. Canine oocytes only expressed *ZP2*, *ZP3* and *ZP4*. The expression levels of *ZP2, ZP3* and *ZP4* were equivalent in IVMCK and IVMP4 oocytes (Figure 3c).

The KEGG pathway analysis compared the IVMCK and IVMP4 groups, and showed DEGs enriched in pathways for certain diseases (graft-versus-host disease, prion disease, ferroptosis, type I diabetes mellitus, Huntington disease and Parkinson disease) and the processing of genetic information, such as RNA transport, RNA degradation, the cell cycle, cholesterol metabolism, ribosomes and spliceosomes (Figure S2a). With regard to cellular components, DEGs in the IVMCK and IVMP4 groups were enriched in GO terms, such as the extracellular region, extracellular exosome, extracellular space and external side of the plasma membrane (Figure S2b). With regards to molecular function, the GO analysis showed that DEGs in the IVMP4 group were enriched in pathways, such as ATPase activity, enzyme activator activity, RNA binding and ATP binding, compared with those in the IVMCK group (Figure S2c). With regards to biological process, DEGs were enriched in the cholesterol metabolic process, embryonic morphogenesis and nucleosome assembly (Figure S2d).

## 4. Discussion

Dogs are an ideal large animal model for studying human diseases, but the preparation of the canine model is limited by ART. In contrast to many other mammals, the maturation of canine oocytes has unique features that have complicated the development of in vitro maturation methods to date. In this study, we successfully acquired the first IVM-IVF-derived dogs by using an optimized medium containing 40 µg/mL P_4_, which may lead to low-cost application of ART and new research avenues for canine species. We attribute this success to two points. First, the concentration of 40 µg/mL P_4_ may have provided an appropriate environment for oocyte maturation. Second, the recipient bitches at the estrus stage were suitable for embryo transfer. Results from the RNA-seq indicated that P_4_ improved embryonic quality. Therefore, high-quality oocytes and the appropriate environment most likely contributed to the live birth of IVM-IVF-derived puppies.

P_4_ is essential for the whole procedure of in vivo maturation of canine oocytes [17]. In most mammals, except for canine species, oocytes complete MII before ovulation [34]. During this period, P_4_ is the major component of follicular fluid in preovulatory follicles, whereas estradiol decreases before the luteinizing hormone surge [35]. In canine species, oocytes complete the MII stage in the oviducts. The canine oviducts also have high concentrations of P_4_, which may contribute to the maturation of oocytes. Although mammalian oocytes have no PR, PR expressed in CCs may contribute to oocyte meiosis [36]. Therefore, P_4_ may directly or indirectly participate in the maturation of oocytes. In several studies, different concentrations of P_4_ were added to an in vitro maturation medium to increase the MII rate of canine oocytes, however, the concentration of P_4_ that had a positive effect were different in published studies. Kim et al. found that 1.0 or 2.0 µg/mL P_4_ produced the highest rate of MII development (10.0% and 10.8%, respectively) compared with other groups (0 and 0.5 µg/mL) [37]. Vannucchi et al. reported that 20 µg/mL P_4_ had a positive effect on GV breakdown but produced a low rate of MII oocytes (<10%) [19]. Another study examined 20–8000 ng/mL P_4_ supplemented into an in vitro maturation medium and showed that more oocytes reached the MII phase with 200 ng/mL P_4_ (10.7%), and concentrations >2000 ng/mL P_4_ reduced canine oocyte maturation [38]. These effects of P_4_ may be related to the improvement of culture systems and successful transplantation. In the present study, we added 10, 40, 100 and 200 µg/mL P_4_ into a basic in vitro maturation medium. The highest maturation rate (29.67%) for dog oocytes was obtained with 40 µg/mL P_4_. Although this concentration was higher than that in the control group, the maturation rate was still low. According to the study of Kim [39], highest number of mature oocytes collected from oocyte donors with serum P_4_ concentrations of 40–50 ng/mL. Previous studies also showed that the P_4_ concentrations in early maturation of oocytes in follicles is approximately 1000 times higher than that of plasma [15], and meiotic resumption of dog oocytes was completed in the oviduct when the P_4_ concentration of plasma reached 20–40 ng/mL [16]. Moreover, the level of P_4_ is always high during the maturation process. Therefore, we speculate that when the level of P_4_ in plasma reaches 40–50 ng/mL, there will be 40–50 µg/mL in the microenvironment of oocytes maturation. Therefore, 40–50 µg/mL supplementation of P_4_ in the culture medium is closest to the mature environment in vivo, which is beneficial for the IVM of canine oocytes.

Fertility and embryonic development were assessed to determine whether the addition of P_4_ had a positive effect on oocyte maturation in vitro. IVV oocytes served as a control group for comparison. IVM oocytes with 40 µg/mL P_4_ had a lower fertilization rate than IVV oocytes (75.9% vs. 89.7%). Meanwhile, the fertilization rate in the 40 µg/mL group (75.9%, 22/29) was higher than that reported previously in dogs (25.9%, 53/205; 28.8%, 30/104) [40,41]. This discrepancy between studies may be attributed to different culture media, and the quality of oocytes and sperm. The findings indicated that 40 µg/mL P_4_ exerted a beneficial effect not only during in vitro maturation, but also at post-fertilization.

Regarding preimplantation embryo development of canine oocytes and the birth rate, a similar trend was observed when we calculated the percentage of the two-cell and four- to eight-cell stages. The number of eight-cell-stage embryos in the IVV group was higher than that in the IVMP4 group, which indicated the low ability of embryonic development. With regard to the birth rate, three living puppies were produced in the IVMP4 group. This is the first report of live puppies being born by in vitro maturation and in vitro fertilization. However, this rate was still low compared with that in the IVV group and it needs to be optimized.

To investigate how P_4_ acts on the maturation of canine oocytes, scRNA-seq was applied to determine changes in gene expression during maturation. IVMP4 oocytes had fewer DEGs than IVMCK oocytes, and the DEGs between IVMP4 and IVV oocytes were tightly clustered. Therefore, the addition of P_4_ allowed IVM oocytes to exhibit transcript expression patterns closer to that of mature oocytes in vivo. The K-means analysis showed that IVMP42 and IVMP44 oocytes were clustered in the IVV group (when K = 2). This finding suggested that the observation point of IVM oocytes with 40 µg/mL P_4_ (IVMP42 and IVMP44) was close to that in the IVV group. K-means data also showed that the three groups were separately clustered when K = 3. This finding suggested that there were differences between the groups, and the observation point for P_4_ supplementation was far from that without P_4_ supplementation, which indicated the benefit of adding P_4_ during in vitro maturation of canine oocytes.

Specific genes regulate the early and late stages of canine oocyte maturation. Considerable upregulation of *MAPK1/3*, *SMAD3* and *BMP6* expression was found in canine oocytes derived from bitches in the estrus stage [42]. *GDF9* and *BMP15* stimulate some degree of meiotic development of canine oocytes [43]. Therefore, we compared their transcriptional levels by scRNA-seq. We found no significant differences in the expression of oocyte maturation-related genes among the three groups, except for *GDF9* and *BMP6*, possibly reflecting the presence of mature oocytes in all samples. The expression levels of *BMP15*, *MAPK1*, *MAPK3*, *SMAD2* and *SMAD3* in IVMP4 oocytes were higher than those in IVV and IVMCK oocytes. These genes are crucial for the MAPK signaling pathway or the transcription of key genes, possibly explaining the success of live births following IVF. The expression levels of *GDF9* and *BMP6* were higher in IVV oocytes than in IVMCK and IVMP4 oocytes. *GDF9* controls the overall process of folliculogenesis, oogenesis and ovulation [44], and *BMP6* controls the initial stage of embryogenesis. Therefore, elevated expression of these factors may have contributed to the low rate of MII stage oocytes in the IVMCK and IVMP4 groups. Additionally, we investigated the expression of oocyte-specific genes affecting early embryonic development. *ZAR1* and *SPIN1* expression levels were higher in IVMP4 oocytes than in IVMCK oocytes, but no difference in expression was found in *NPM2* or *HSF1*. *HSF1* normally controls early post-fertilization development in mice [45] and the initial development of oocytes [46]. *ZAR1* was the first identified oocyte-specific maternal effect gene that functions at the oocyte-to-embryo transition [47]. *NPM2* is a maternal effect gene that is critical for nuclear and nucleolar organization and embryonic development [48]. *SPIN1* plays a role in cell cycle regulation during gamete-to-embryo transition [49]. These results indicate that P_4_ supplementation is beneficial for the early development of canine embryos and support the conclusion that P_4_ improves the maturation quality of canine oocytes.

We also evaluated the fertilization capabilities of canine oocytes. The ZP can help oocytes exchange nutrients, metabolites, and other molecules within the extracellular environment, and promote oocyte growth and follicular expansion [50]. ZP proteins provide a site for sperm to bind and undergo the acrosome reaction, allowing sperm to penetrate the ZP to fertilize oocytes. There are three ZP proteins in canine oocytes called ZP2, ZP3 and ZP4. ZP3 is recognized as the primary sperm receptor [51], whereas ZP2 acts as a secondary sperm receptor that helps sperm adhesion to the ZP [52]. In humans, ZP4 (ZP1-like protein) induces the acrosome reaction and binds to capacitated acrosome-intact spermatozoa [53]. Oocytes of *ZP2*- or *ZP3*-knockdown mice have weak developmental capacity and produce no offspring [54,55]. In our study, scRNA-seq showed that expression levels of *ZP2*, *ZP3* and *ZP4* were equivalent in IVMCK and IVMP4 oocytes. A previous study showed that P_4_ regulated the expression of ZP glycoproteins in a dose-dependent manner [56], but 2 µg/mL P_4_ was the highest concentration examined. How 40 µg/mL P_4_ affects the expression of ZP genes is unclear.

In this study, the enriched KEGG pathways for DEGs between IVMCK and IVMP4 oocytes were mainly related to certain diseases and the processing of genetic information. This finding suggests that an important difference between these two groups may involve the transmission of genetic information, such as ribosome translation function and oxidative phosphorylation. Although the exact mechanism of P_4_ actions in dog oocytes remains unclear, previous studies have indicated that it involves an immediate, but transient, decrease in cyclic AMP (cAMP) [56,57] and the activation of Aurora A [58], which is a member of the Aurora family of protein kinases. In the current study, adding P_4_ may have increased the maturation rate by binding to a specific receptor (still unknown) to increase the level of guanine nucleotide-binding protein G(i) subunit alpha-2 and reduce the cAMP concentration. Mitotic regulators, such as *MAP2K1*, kinesin family member 22 [59] and serine/threonine-protein kinase 1, were highly expressed in IVMP4 oocytes, which suggested that P_4_ was beneficial for the maturation and subsequent development of canine oocytes (Figure S3). Transcripts associated with GO analysis terms, such as cellular component, molecular function and biological process, also indicated that P_4_ was helpful for later embryonic development.

To the best of our knowledge, this is the first report to show that puppies can survive by IVM and IVF technology. This finding suggests that IVM canine oocytes can subsequently develop in vivo. To date, two puppies are still alive with no detectable difference between puppies born from a normal mating, and one died of a disease.

In summary, we obtained high-quality IVM dog oocytes that enabled the production of IVF-derived puppies. This advance provides a new opportunity for easy and cost-efficient ART for canines. Adding 40 µg/mL P_4_ significantly increases the maturation rate of IVM canine oocytes, and scRNA-seq suggests that P_4_ supplementation also improves their developmental potential. However, the exact pathway still needs to be mapped, and the IVM system needs further modifications. Overall, our research led to the establishment of a general method for the production of canine embryos using IVM oocytes. This method could provide a promising foundation for more extensive application to develop canine somatic cell nuclear transfer models, and for the preservation of endangered canines and creation of canine disease models.

## Figures and Tables

**Table 1:** Nuclear maturation rate of canine cumulus–oocyte complexes with different concentrations of progesterone (P_4_).

P_4_ Concentration (μg/mL)	No. of Oocytes Examined	No. of Oocytes at Various Meiotic Stages				
		GV%	GVBD%	MI%	MII%	DE%
0	118	3.8 ± 3.3	6.2 ± 5.6	19.4 ± 12.8	13.2 ± 2.1 ^bc^	57.5 ± 5.4
10	71	10.8 ± 4.1	17.4 ± 4.3	19.7 ± 11.2	20.4 ± 2.7 ^b^	29.7 ± 1.4
40	90	9.9 ± 2.7	10.7 ± 6.1	21.9 ± 6.7	29.7 ± 7.1 ^a^	27.8 ± 7.3
100	84	16.7 ± 7.3	18.0 ± 7.0	21.7 ± 8.8	12.3 ± 4.0 ^bc^	30.9 ± 5.7
200	224	8.7 ± 4.7	12.0 ± 6.8	25.7 ± 7.0	11.5 ± 7.7 ^c^	44.9 ± 5.0

Data for germinal vesicle (GV), germinal vesicle breakdown (GVBD), metaphase I (MI), metaphase II (MII) and degenerated (DE) oocytes are shown. Data are shown as the mean ± standard deviation. Different letters in columns indicate significant differences between different treatment groups (*p* < 0.05). The same letter in the same column indicates no significant difference between the treatments (*p* > 0.05).

**Table 2:** Comparison of development of canine oocytes after in vitro fertilization.

Group	Treatment (*n*)	Cleavage (*n*)	2-Cell (*n*)	4-Cell to 8-Cell (*n*)	8-Cell (*n*)
In vivo	29	26	6	13	7
In vitro (40 μg/mL P_4_)	29	22	6	12	4

**Table 3:** Embryo transfer of canine oocytes after in vitro fertilization.

Group	Stage	Transplant Location	Embryo (*n*)	Pregnant (25 d) (*n*)	Birth (*n*)
In vivo	2-8 cell	oviduct	7	6	6
	2-4 cell	oviduct	2	0	0
In vitro	2-4 cell	oviduct	6	0	0
	2-8 cell	oviduct	10	4	3

**Table 4:** Number of differentially expressed genes in IVMP4 oocytes, IVMCK oocytes, and IVV oocytes.

A_vs_B	NO_DEG	NO_DEG_UpInA	NO_DEG_UpInB
IVMCK_vs_IVV	4366	2365	2000
IVMP4_vs_IVMCK	1233	701	531
IVMP4_vs_IVV	2590	1443	1146

A_vs_B: comparison between sample A and sample B; NO_DEG: number of differentially expressed genes between samples A and B; NO_DEG_UpInA: number of differentially expressed genes upregulated in sample A; NO_DEG_UpInB: number of differentially expressed genes upregulated in sample B. IVMP4, in vitro matured with progesterone; IVMCK, in vitro matured without progesterone; IVV, in vivo matured.

## Data Availability

Data supporting the results are available from the correspondence author upon reasonable request.

## Supplementary Materials

The following supporting information can be downloaded at: https://www.mdpi.com/article/10.3390/life12111778/s1. Figure S1: Different stages of in vitro maturated—in vitro fertilized canine oocytes. Figure S2: KEGG pathway and Gene Ontogeny analysis of oocytes matured in vitro with or without progesterone; Figure S3: Simplified progesterone-mediated oocyte maturation pathway. Table S1: Summary of sequencing quality by samples; Table S2: STR analysis of IVF dogs and surrogates.
