# Supplementary material for: Progesterone Promotes In Vitro Maturation of Domestic Dog Oocytes Leading to Successful Live Births
Source: Life (Basel). 2022 Nov 3;12(11):1778. doi: 10.3390/life12111778 (PMC9698205; doi:10.3390/life12111778)
Supplement: Supplementary file 1 [file life-12-01778-s001.zip › life-1951140-supplementary/Supplementary Table.pdf]

Supplementary Table

Table S1. Summary of sequencing quality by samples

SampleID	RawReads	Q30	Q20	GC
IVMCK2	23491041,23491041	92.77%,90.36%	97.03%,95.83%	45.83%,45.95%
IVMCK3	22677072,22677072	93.06%,90.00%	97.16%,95.66%	44.92%,45.09%
IVMCK4	25459055,25459055	92.91%,90.08%	97.01%,95.61%	45.11%,45.27%
IVMP42	23998233,23998233	93.06%,90.33%	97.15%,95.83%	44.20%,44.37%
IVMP43	24788608,24788608	92.48%,89.80%	96.79%,95.45%	44.81%,45.00%
IVMP44	23365797,23365797	93.30%,90.72%	97.34%,96.06%	45.76%,45.88%
IVV2	44727962,44727962	94.20%,93.86%	97.38%,96.97%	44.80%,44.74%
IVV3	34422853,34422853	93.91%,93.16%	97.19%,96.58%	44.45%,44.44%
IVV4	35332377,35332377	94.10%,93.35%	97.34%,96.73%	44.63%,44.63%

RawReads : Total number of sequenced raw reads; Q30 : Percentage of nucleotides passed Q30 (Q30: error rate=1/1000); Q20 : Percentage of nucleotides passed Q20 (Q20: error rate=1/100); GC : GC : Percentage of GC content

Table S2. STR analysis of IVF dogs and surrogates

Number / Site	6 （Male)	586 （Surrogate)	Milk （IVF puppies)	Small Black （IVF puppies)	Yellow （IVF puppies)
PEZ1	120/120	116/120	116/120	116/120	116/120
PEZ2	175/183	167/179	183/183	183/183	175/183
FH2010	215/215	219/223	215/223	215/219	215/223
PEZ5	262/266	266/266	262/266	262/266	262/266
PEZ12	308/308	308/308	308/308	308/316	308/308
PEZ21	140/140	132/140	140/140	132/140	140/144
PEZ3	185/191	185/188	182/191	191/191	182/185
PEZ6	276/276	263/263	267/276	-	267/276
PEZ8	351/354	340/344	340/354	354/358	351/362
FH2054	122/134	122/130	122/142	134/142	122/142
VWFX	192/192	186/192	192/198	186/192	192/198
FH2611	236/236	244/251	238/238	248/248	236/238
FH2132	282/378	290/294	338/378	-	378/382
PEZ20	116/124	124/128	116/124	120/124	116/120
PEZ15	186/193	190/193	183/210	193/210	186/210
FH2079	263/263	263/263	263/263	263/263	263/263
DA	X/Y	X/X	X/X	X/Y	X/Y
