# Supplementary figures and images for: Progesterone Promotes In Vitro Maturation of Domestic Dog Oocytes Leading to Successful Live Births
Source: Life (Basel). 2022 Nov 3;12(11):1778. doi: 10.3390/life12111778 (PMC9698205; doi:10.3390/life12111778)

Figure S1

a

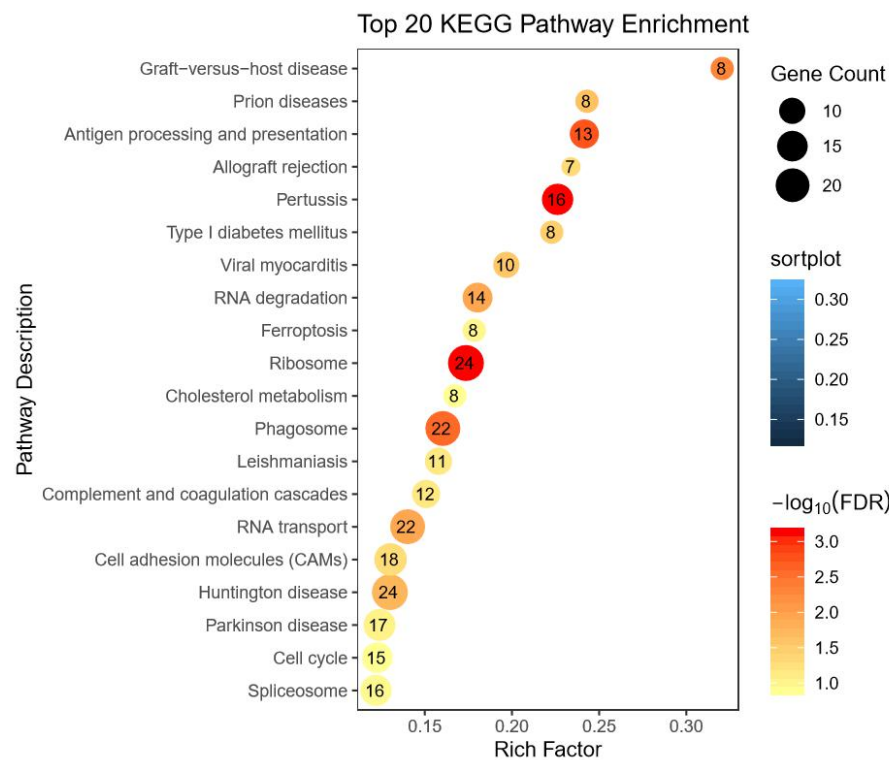

b

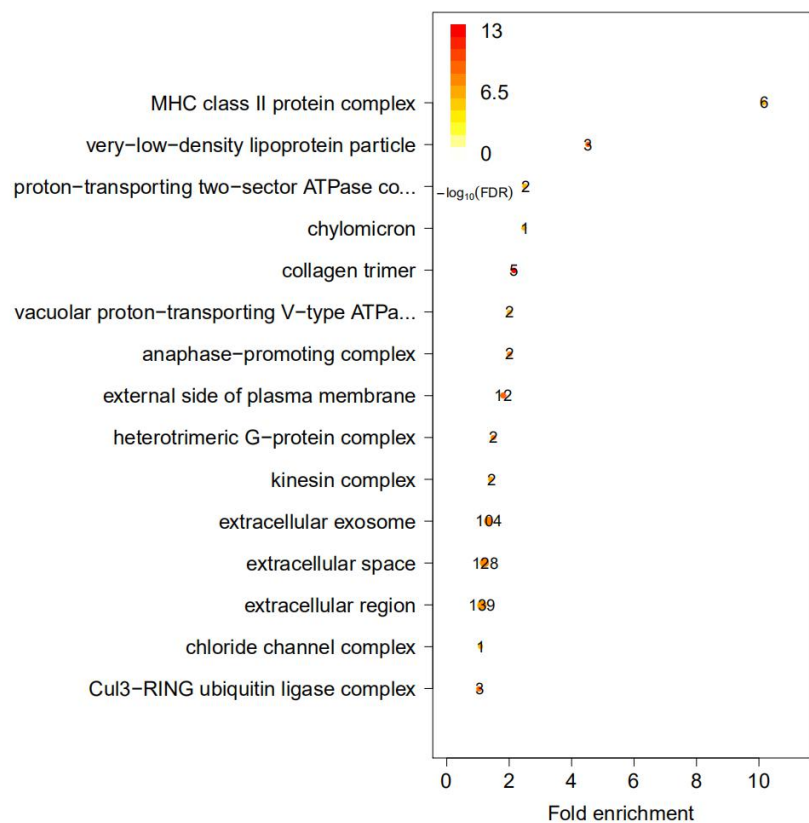

c

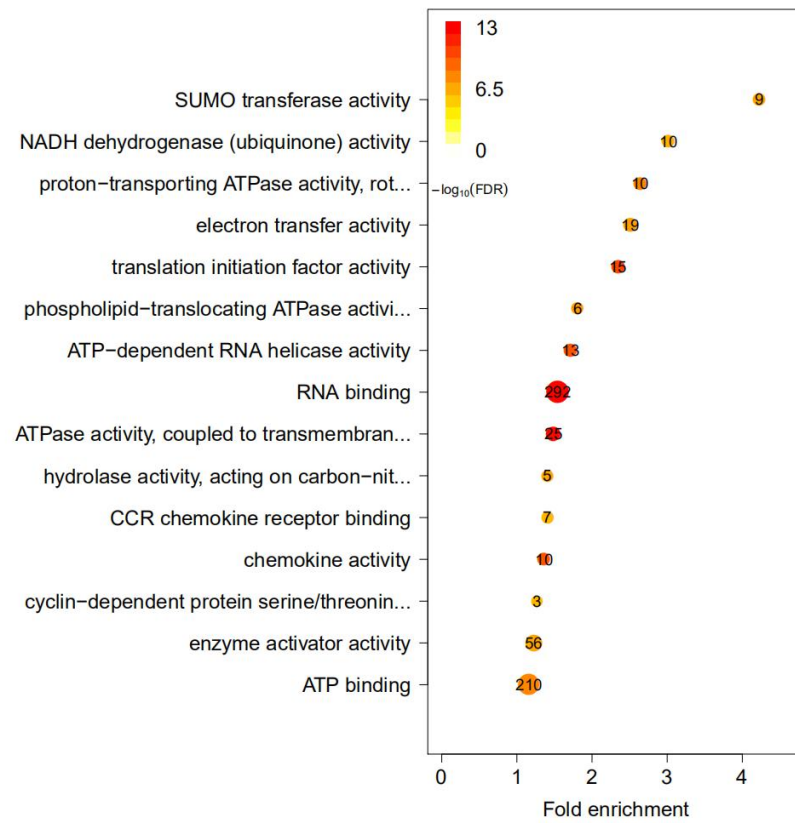

d

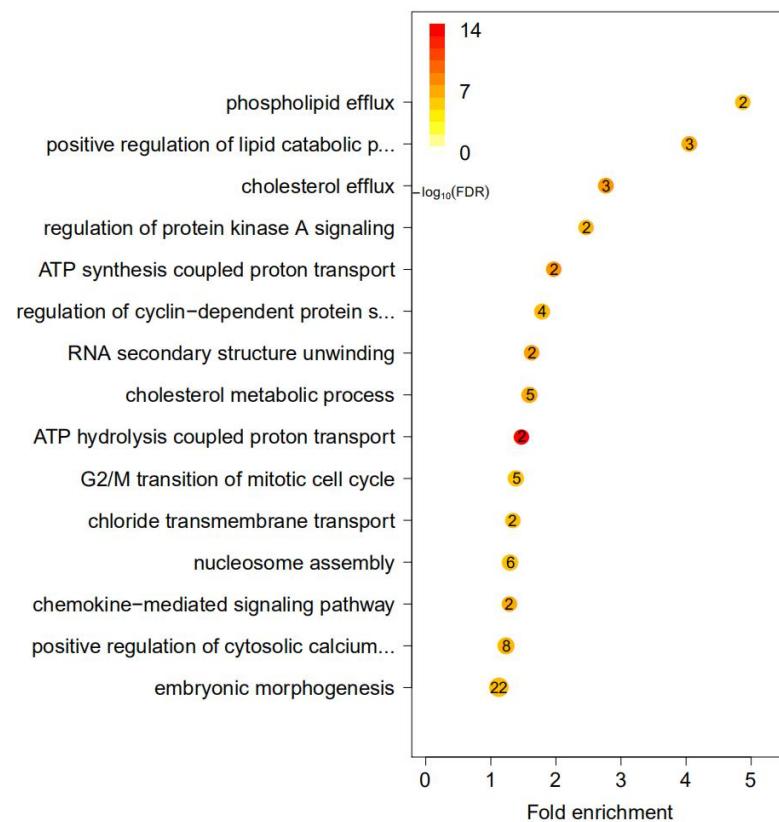

Supplement: Supplementary file 1 [file life-12-01778-s001.zip › life-1951140-supplementary/Figure S1.pdf]

Figure S2

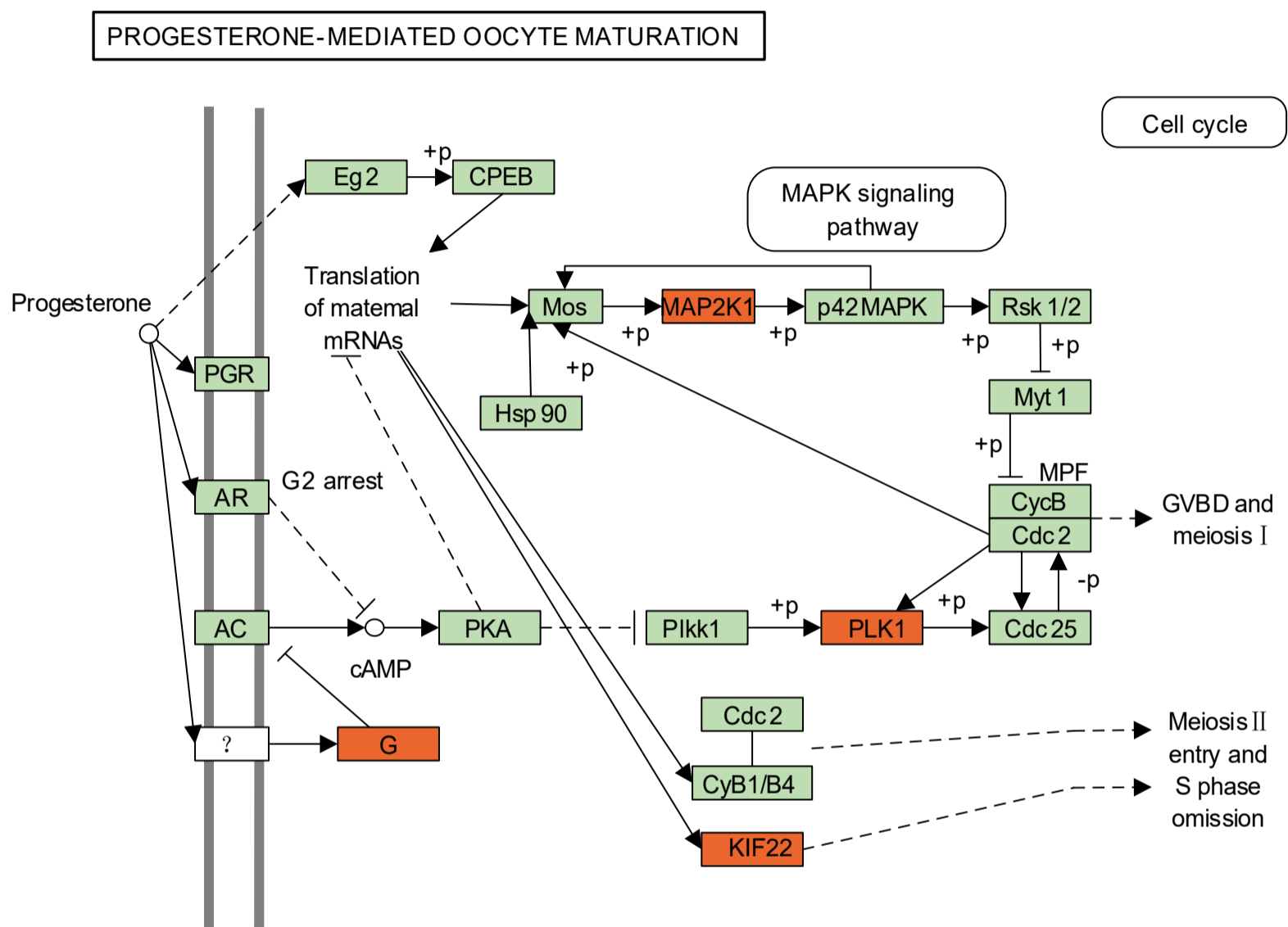

Supplement: Supplementary file 1 [file life-12-01778-s001.zip › life-1951140-supplementary/Figure S2.pdf]
